# Superior mesenteric vein tumour thrombus in a patient with caecal adenocarcinoma: a rare and important finding

**DOI:** 10.1259/bjrcr.20200147

**Published:** 2021-01-05

**Authors:** Janki Trivedi, Heinrich Bouwer, Tom Sutherland

**Affiliations:** 1 St Vincent’s Hospital Melbourne, Victoria, Australia; 2 Victorian Institute of Forensic Medicine, Southbank, Australia; 3 Department of Forensic Medicine, Monash University, Melbourne, Australia; 4 Faculty of Medicine, University of Melbourne, Victoria, Australia

## Abstract

Venous tumour thrombosis refers to the invasion of tumour into the venous system. Extramural venous invasion is routinely searched for and reported in rectal carcinoma due to its prognostic significance and influence on staging, prognosis and treatment approach. We describe a case of extramural venous invasion occurring as superior mesenteric vein tumour thrombus in the setting of a caecal carcinoma.

## Clinical presentation

A 45-year-old female with alcohol dependence and difficult social circumstances was brought by paramedics to the emergency department (ED) in an intoxicated state due to decreased Glasgow coma scale (GCS). Preliminary blood tests revealed elevated lactate at 3.8 mmol l^−1^ (reference range 0.5–1.6 mmol l^−1^) and anaemia with a haemoglobin (Hb) of 85 g l^−1^ (reference range 115–165 g l^−1^) which had dropped from 116 g l^−1^ 1 month prior.

In the context of the non-specific abdominal tenderness and Hb drop, a contrast-enhanced CT abdomen and pelvis was performed which did not reveal any acute intra-abdominal pathology. There was also no evidence of bowel oedema or ischaemia. The patient was being managed by her general practitioner for iron-deficiency anaemia with regular iron transfusions. After overnight observation, the patient was asymptomatic and discharged home.

Around 2 months after this, the patient represented to the ED in an intoxicated state complaining of abdominal pain with Hb 80 g l^−1^. A digital rectal examination revealed melena. B12, folate studies and pelvic ultrasound were ordered and were normal. Iron studies revealed ferritin was at the lower limits of normal at 37 g l^−1^ (reference range 30–200), low iron of 3 mol l^−1^ (reference range 9.0–30.4 mol l^−1^), transferrin 2.7 g l^−1^ (reference range 2.0–3.6) and very low transferrin saturation of 4% (reference range 20–50%). A CT scan was not performed during this admission. The patient was referred to gastroenterology with a plan for outpatient colonoscopy.

## Investigations

Initial colonoscopy booking was not attended due to complicated psychosocial factors and over this period, there were multiple repeated emergency presentations for alcohol intoxication and symptomatic anaemia.

A subsequent colonoscopy and biopsy performed 4 months after the initial ED presentation revealed a non-obstructing caecal adenocarcinoma occupying one-third of the lumen circumference measuring 5 cm in length.

**Figure 1.**
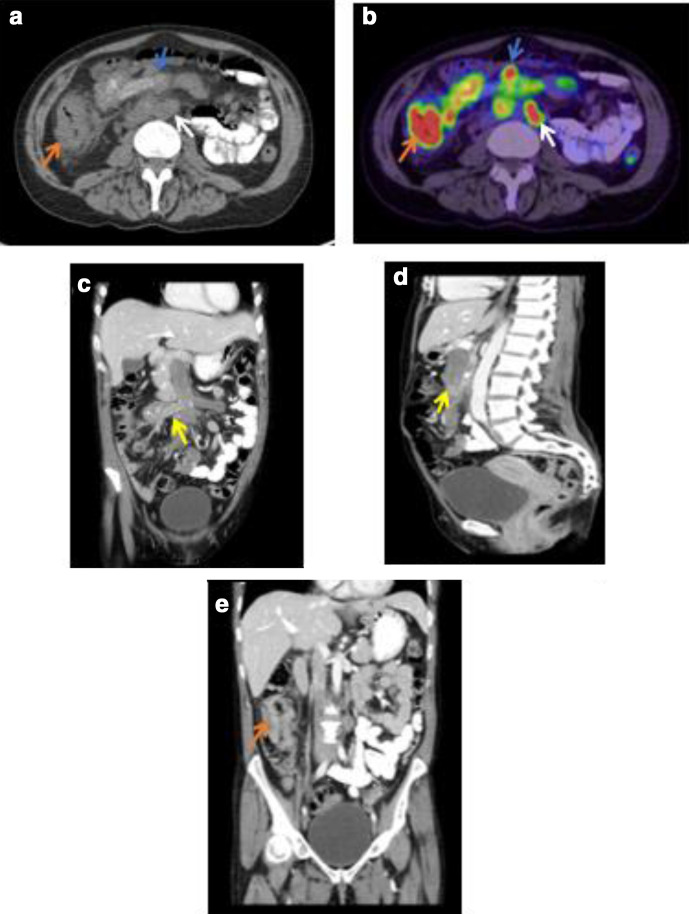
(a-e) Staging (a) Axial CT and (b) FDG-PET CT images showing intensely FDG-PET avid primary caecal tumour (red arrow) and partially calcified tumour thrombus in the SMV (blue arrows) extending into tributaries. Para-aortic nodal metastases are noted (white arrows). (c) Coronal and (d) Sagittal CT images confirm the extensive venous thrombus (yellow arrows) and (e) shows the caecal mass (red arrow). Bland thrombus is most likely to be on the downstream tip of tumour thrombus. FDG, Fludeoxyglucose; PET, Positron emission tomography; SMV, Superior mesenteric vein.

**Figure 2.**
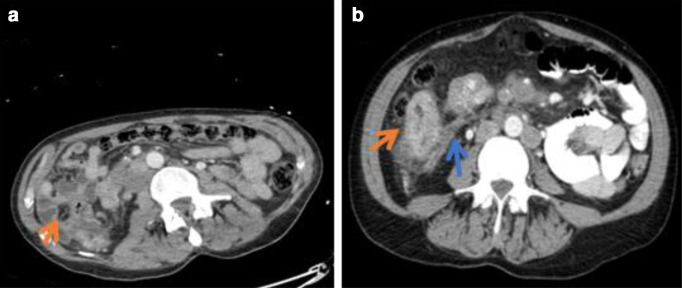
(a) Axial contrast-enhanced CT abdomen showing normal non-dilated bowel loops in the lower quadrant at the level of the caecum (red arrow). (b) Axial contrast-enhanced CT abdomen performed 4 months after (a) shows significant thickening of the caecum (red arrow) with circumferential luminal narrowing and extensive surrounding fat stranding and mesenteric invasion along the medial aspect of the caecal mass (blue arrow).

The patient’s haemoglobin declined to 80 g l^−1^ at the time of biopsy. A staging CT and FDG-PET (fludeoxyglucose-positron emission tomography) confirmed multiple enlarged para-aortic nodes and extensive tumour invasion in the superior mesenteric vein (SMV) passing the level of the caecal tumour to the origin of the portal vein with intense metabolic activity within the primary tumour (SUV (max) 22.4) and SMV (SUV (max) 11.8) (Figure 1). The tumour thrombus in the smaller peripheral ileocolic vein contained mottled calcification while the more central venous involvement was free of calcification. There was no evidence of these findings upon review of the initial CT (Figure 2).

## Treatment

The patient was planned for urgent systemic chemotherapy with an aim to downsize the tumour and potential surgical resection. In the interim, the patient developed melena and worsening abdominal pain with anaemia and was administered regular iron infusions and blood transfusions. She also developed desaturations and a CT angiography of the pulmonary arteries was performed which showed bilateral subsegmental pulmonary emboli and she was managed with therapeutic Enoxaparin.

## Outcome and follow-up

Prior to commencing chemotherapy, the patient was admitted to undertake alcohol detoxication. Unfortunately, during this period and prior to initiating chemotherapy, the patient was found deceased on her couch with post-mortem examination and toxicology finding of mixed drug toxicity as the cause of death.

**Figure 3.**
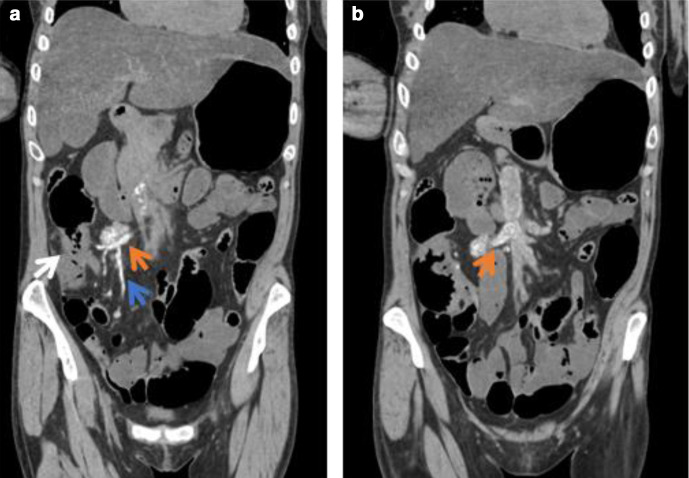
(a, b) Post-mortem CT coronal images (2 months post-CT in Figure 1) showing increased calcification in the SMV tumour thrombus (red arrows) and extending into tributaries (blue arrow). The caecal mass causing near complete luminal obstruction is also noted (white arrow). SMV, Superior mesenteric vein.

A post-mortem CT showed that the extent of tumour thrombus was similar to the pre-mortem staging imaging of 2 months earlier; however, the degree of calcification had increased (Figure 3).

## Discussion

Venous tumour thrombosis can be seen in patients with abdominal malignancies such as renal cell carcinoma, adrenal cortical carcinoma, hepatocellular carcinoma, gastric or pancreatic carcinoma. The incidence of venous tumour thrombosis in colorectal cancer, however, is rare with Sato et al in 2010 reporting venous tumour thrombosis only in 1.7% (3/176) patients with advanced colorectal cancer.^1,2^

Accurate diagnosis of the nature of the portal vein thrombus, related to thromboembolism or tumour thrombus, is extremely important to guide management. This can avoid unnecessary anticoagulation as would be the case to manage a benign or bland thrombus.

The reported incidence of macroscopic tumour thrombus is very low at approximately 2.8%.^3^ This case highlights a caecal adenocarcinoma with pathologically confirmed venous tumour thrombus. Given that blood clot thrombosis and tumour thrombosis both have low-attenuation; it can be difficult to distinguish on the basis of CT alone. In general, bland thrombus is non-enhancing on imaging while tumour thrombus is enhancing and heterogeneous enhancement is generally described.

**Figure 4.**
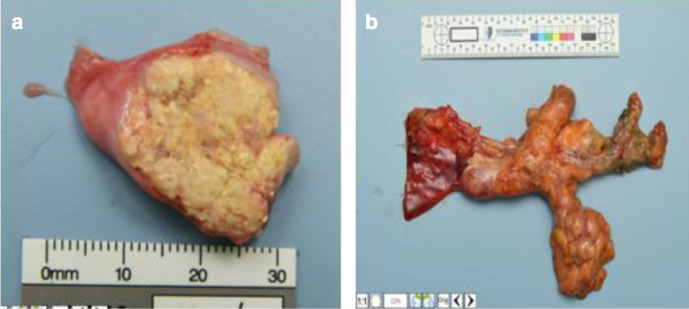
(Aa b) Pathological axial cross-section specimen images showing infiltration of the SMV by macroscopic tumour confirmed as adenocarcinoma. SMV, Superior mesenteric vein.

As in our case, FDG-PET is useful to confirm tumour thrombus which would demonstrate intense FDG activity like the primary tumour site. However, the SUV can be unreliable in small tumours such as in the vein especially when calcification exists. Increased cross-sectional size of the vessel due to expansion is a very important factor in diagnosing tumour thrombus with confidence. Direct continuity from the primary tumour to the involved veins and shared imaging characteristics is also useful in making the correct diagnosis. Bland thrombus can enhance occasionally as it begins to recanalise, and thus as such no single feature is 100% specific and these features need to be considered overall in the specific clinical scenario. In our case, it can be presumed that the anticoagulation treated the bland component of the thrombus and the remainder was tumour thrombus, and this is consistent with the post-mortem histology finding of metastatic tumour thrombus (Figure 4).

Calcification within tumour thrombus is rare and not commonly reported in current literature but calcification within the tumour itself has been frequently described in abdominal malignancies like colorectal and ovarian cancers.^4^ Mesenchymal, lymphoid and germ cell tumours can also calcify.^4^ Metastatic calcification occurs in the setting of hypercalcaemia usually due to hyperparathyroidism and often secondary to renal dysfunction.^4^ The mucinous subtype in colorectal cancer is most likely to calcify and also carries a worse prognosis.^4^ Easson et al^5^ also noted that some of the noncalcified colorectal metastases to the liver developed calcifications during therapy; however, they could find no association with chemotherapeutic agents or response to therapy.^5^

In their review of 43 cases, Otani et al^1^ suggested that patients with colorectal cancer and venous tumour thrombus are generally a cohort with more aggressive primary cancer and are at greater risk of developing liver metastasis.^1^ In their meta-analysis, complete tumour resection was possible in 32 (74.4%) of 43 patients with colon cancer and venous tumour thrombosis.^1^ In addition to being associated with a poor prognosis, venous tumour invasion also has implications for planning primary tumour surgical resection and whether it is amenable to thrombectomy and grafting.^6^ It also influences the adjuvant chemotherapy regime and duration which likely has to be extended in the presence of venous tumour invasion.^7,8^ Although in our case, the patient was planned for aggressive adjuvant chemotherapy and consideration of surgery, the complex psychosocial factors and alcohol intoxication caused delays in diagnosis and management and this contributed to an unfortunate outcome.

This case highlights the importance of searching for venous invasion in colonic carcinomas as a marker of aggression and metastatic potential.

## Learning points

- Closely assessing the SMV and tributaries is important on all imaging for patients with known colorectal cancer.
- PET/CT is useful in differentiating blood clot venous thrombus from venous tumour thrombus.
- SMV invasion by tumour thrombus can significantly alter the patient’s oncological and surgical management.
- There is a high incidence of liver metastasis with venous tumour thrombosis which can impact on the patient’s prognosis.
